# Elevated peripheral glutamate and upregulated expression of NMDA receptor NR1 subunit in insomnia disorder

**DOI:** 10.3389/fpsyt.2024.1436024

**Published:** 2024-10-07

**Authors:** Jingjing Lin, Xiaohui Hou, Yaxi Liu, Yixian Cai, Jiyang Pan, Jiwu Liao

**Affiliations:** ^1^ Department of Psychiatry, The First Affiliated Hospital of Jinan University, Guangzhou, Guangdong, China; ^2^ Department of Psychiatry, Sleep Medicine Centre, The First Affiliated Hospital of Jinan University, Guangzhou, Guangdong, China

**Keywords:** glutamate, glutamine, N-methyl-D-aspartate, NR1 subunit, insomnia disorder

## Abstract

**Background:**

The present study explored the serum glutamate (Glu), glutamine (Gln), glutamic acid dehydrogenase (GAD) concentrations and the mRNA expression levels of the N-methyl-D-aspartate receptor (NMDAR) NR1 subunit in the peripheral blood of patients with insomnia disorder (ID). To our knowledge, this is the first study showing an increase in the mRNA expression levels of the NMDAR NR1 subunit in patients with ID.

**Methods:**

This study included 30 ID patients and 30 matched healthy controls. We investigated the demographic and illness information and assessed subjective sleep quality using the Pittsburgh Sleep Quality Index. The Hamilton Depression Scale-17 and Hamilton Anxiety Scale were used to evaluate the patients’ symptoms of depression and anxiety, respectively. The quantifications of Glu, Gln and GAD concentrations were performed by Enzyme-linked immunosorbent assay (ELISA). Real-time PCR was used to detect the mRNA expression levels of the NMDAR NR1 subunit in peripheral blood.

**Results:**

Compared with the healthy control group, the serum Glu concentrations and the mRNA expression levels of the NMDAR NR1 subunit in the ID group were significantly higher. However, there was no significant difference in Gln and GAD between the two groups. The receiver operating characteristic (ROC) analysis showed that the mRNA expression levels of the NMDAR NR1 subunit could distinguish ID patients from healthy individuals (area under the curve: 0.758; sensitivity: 73.3%; specificity: 76.7%). A negative correlation was found between the mRNA expression levels of the NMDAR NR1 subunit for age, total duration of illness, and age of first onset in the ID group, whereas a positive correlation was detected for daytime dysfunction.

**Conclusion:**

Glutamatergic neurotransmission was abnormal in ID patients. Additionally, the mRNA expression levels of the NMDAR NR1 subunit appeared to have potential as a clinical biomarker for ID. However, the sample size of our study was limited, and future studies with larger sample sizes are needed to further validate and explore the mechanisms involved and to assess the reliability of the biomarker.

## 1 Introduction

Insomnia disorder (ID) is the most prevalent sleep disorder, characterized by challenges in initiating or maintaining sleep and early morning awakenings accompanied by an inability to return to sleep. These sleep problems frequently occur despite adequate opportunities for sleep and cause daytime impairment (1). With a global prevalence of 19%–50% among adults, ID causes a heavy economic burden and conveys increased risks for various diseases, such as cardiovascular disease, diabetes, depression, and anxiety (2–6). Studies have demonstrated that ID is influenced by heritability, adverse experiences, lifestyle, and other factors (7, 8). However, the pathogenesis of ID is not entirely understood and requires further investigation. ID diagnosis has been mainly based on self-reported sleep difficulties, and no perfect diagnostic marker for ID has been identified.

Glutamate (Glu) is a primary excitatory neurotransmitter in the central nervous system (CNS) and peripheral organs. Existing research has reported changes in Glu levels across various mental disorders (9–11). Moreover, Glu regulates spontaneous and rhythmic electrical neuron activity and participates in sleep and wakefulness (12–14). Activation of glutamatergic neurons induces arousals that disrupt sleep continuity (15). Glutamatergic neurons in the parabrachial nucleus serve as the primary source of ascending arousal influence from the brainstem (16). So far, a few studies have used magnetic resonance spectroscopy (MRS) to investigate the changes in brain Glu in ID patients, and the results have been inconsistent (17–19). By contrast, few studies have analyzed the Glu of peripheral blood in ID people.

During synapse activity, astrocytes uptake neuronal Glu, converting it to glutamine (Gln) or oxidizing it through the tricarboxylic acid cycle. The Gln is subsequently transported back to neurons and reconverted to Glu. This continuous process, from the release of neuronal Glu to the regeneration of Glu from Gln, is referred to as the Glu–Gln cycle (20). Another metabolic route for Glu entails its transformation into Gamma-aminobutyric acid (GABA) through the catalysis of glutamic acid decarboxylase (GAD) (21). Gln plays a crucial role within the brain as a non-neuroactive intermediary in the recycling process of amino acid neurotransmitters, primarily Glu and GABA (22). To date, little evidence has been found associating GAD with ID. Several studies have employed MRS to explore alterations in brain Gln among ID patients, yielding inconsistent findings. Glu/Gln (Glx) levels may reflect hyperarousal at bedtime in ID patients (23). An MRS-based study found that Glx levels did not differ between those with shorter and longer sleep duration (24).

Glu is synthesized mainly in glutamatergic neurons and released from the presynaptic axon termini into the synaptic gap to activate Glu receptors specifically. Glu receptors are divided into two major groups: a family of metabotropic Glu receptors (mGluR) and ionotropic ligand-gated ion channels, such as N-methyl-D-aspartate receptor (NMDAR). The NR1 (or GluN1) subunit is fundamentally composed of NMDAR (25). Studies have confirmed that NMDAR and its subunits are associated with psychotic disorders (26, 27). Glu increased wakefulness possibly via the action of NMDAR (28). A recent experiment reported that NMDAR in the lateral preoptic hypothalamus is essential for sustaining sleep, and its activation stabilizes the firing of sleep-on neurons (29). Moreover, some selective NMDAR antagonists induce sedation, displaying similarities to the characteristics of natural deep sleep (30).

Despite the blood-brain barrier (BBB), previous studies have proven that metabolism in the CNS may influence and be influenced by peripheral compounds through cytokines, neurotransmitters, or hormones (31). Partial Glu enters the bloodstream from the CNS (32). Moreover, NMDAR is not only present in neuronal cells but also in various non-neuronal cells, such as platelets and immune system cells, suggesting the possibility of common regulatory mechanisms between peripheral cells and neurons (33, 34).

The role of Glu and NMDAR in ID has not been clearly defined. To our knowledge, no study has been conducted in ID patients focusing on the mRNA expression levels of the NMDAR NR1 subunit in peripheral blood. Given the participation of Glu and its receptors in the etiology of sleep, we detected the serum Glu concentrations and the mRNA expression levels of the NMDAR NR1 subunit in peripheral blood. We aimed to identify the potential of serum Glu concentrations and the mRNA expression levels of the NMDAR NR1 subunit as feasible biomarkers for ID, contributing to a better understanding of ID pathophysiology and improving the accuracy of ID diagnoses.

## 2 Materials and methods

### 2.1 Participants

The present study recruited participants diagnosed with ID in the First Affiliated Hospital of Jinan University from March to December 2023.

The inclusion criteria of the ID group were as follows (1): patients who met the Diagnostic and Statistical Manual of Mental Disorders, 5th Edition (DSM-5) diagnostic criteria for insomnia disorder (F51.01) (2); insomnia symptoms had lasted over 3 months (3); patients who aged 18-65 years (4); junior high school or above education background (5); Pittsburgh Sleep Quality Index (PSQI) ≥ 8 points.

The exclusion criteria were as follows (1): combined with other sleep-wake disorders, including obstructive sleep apnea syndrome (apnea–hypopnea index > 15/h) or periodic limb movement of sleep (Periodic Limb Movement during Sleep Index > 15/h), which was determined using overnight polysomnography (PSG) (2); combined with the organic brain, severe somatic, and neurological diseases (3); insomnia symptoms are caused by substance use (4); previous or present diagnoses of mental disorders affecting sleep include anxiety disorders, depression disorders, bipolar disorders, schizophrenia, and post-traumatic stress disorder (5); Hamilton Depression Scale-17 (HAMD-17) > 17 points (6); Hamilton Anxiety Scale (HAMA) ≥ 14 points (7); history of shift work or jetlag (8); history of taking sedative-hypnotics in the past two weeks (9); pregnant and lactating women.

The healthy control (HC) population was recruited in the same period from nearby communities. The inclusion criteria of the HC group were as follows (1): previously or currently did not meet the diagnostic criteria for ID in DSM-5 (2); aged 18-65 years (3); PSQI < 8 points. The exclusion criteria for the HC group were consistent with the aforementioned ID group.

The study was performed at The First Affiliated Hospital of Jinan University with approval from its Medical Ethics Committee (Approval #KY-2023-113). All participants provided written informed consent before participating, and researchers adhered to the Declaration of Helsinki Principles.

### 2.2 Clinical assessment

All participants were assessed based on the Mini International Neuropsychiatric Interview (M.I.N.I.) by two professional psychiatrists to rule out other psychiatric disorders. Meanwhile, the participants underwent overnight PSG to detect other sleep-wake disorders. The demographic information was investigated, including age, gender, marital status (classified into two groups: currently married and not married), education (described as the total years of formal education completed), and family history of ID (whether a clear history of ID existed among the relatives). Moreover, we gathered illness related information in ID patients, encompassing age at first onset, current illness duration, total illness duration, and whether it constituted a first episode. Furthermore, several scales were used in our study including the PSQI, HAMD-17 and HAMA. The PSQI was used to measure self-rated sleep quality and sleep disturbance over a 1-month interval, including subjective sleep quality, sleep latency, sleep duration, habitual sleep efficiency, sleep disturbances, use of sleeping medication, and daytime dysfunction (35). The HAMD-17 and HAMA were used to rate depression and anxiety symptoms in the patients, respectively.

### 2.3 Analysis of Glu, Gln, GAD and NMDAR NR1 subunit

Peripheral venous blood from the participants was drawn in a fasting state in dry tubes and EDTA anticoagulant tubes between 8:00 and 9:00 a.m. the next day after enrollment. The dry tubes (2 mL blood sample/tube) were stored at 4°C for 30 min, followed by low-temperature centrifugation (4°C) at 3000 r/min for 10 min. After centrifugation, the supernatant was transferred to a cryopreservation tube and stored in a refrigerator at –80°C. Glu, Gln and GAD concentrations were determined using an enzyme-linked immunosorbent assay (ELISA) kit (Exodiagnosis Biotechnology Co., Ltd, Guangzhou, China). The concentrations of the standard substance included in the Glu kit were 48, 24, 12, 6, 3, and 0 mg/L. The detection range of the kit was 1.5–48 mg/L, the sensitivity was 0.1 mg/L, the intra-assay coefficient of variation was 5%, and the inter-assay coefficient of variation was 10%. The concentrations of the standard substance included in the Gln kit were 1600, 800, 400, 200, 100 and 0 µmol/L. The detection range of the kit was 50-1600 µmol/L, the sensitivity was 10 µmol/L, the intra-assay coefficient of variation was 6%, and the inter-assay coefficient of variation was 11%. The concentrations of the standard substance included in the GAD kit were 48, 24, 12, 6, 3 and 0 U/L. The detection range of the kit was 1.5-48 U/L, the sensitivity was 0.1 U/L, the intra-assay coefficient of variation was 5%, and the inter-assay coefficient of variation was 10%.

Blood samples in the EDTA anticoagulant tubes were used to detect mRNA expression levels of the NMDAR NR1 subunit. Total RNA was extracted using the Blood Sample RNA Extraction Kit (OMEGA). A purity test was performed: 1 µL of the RNA sample was diluted 50-fold, and the optical density (OD) value was measured with the Biophotometer plus Eppendorf Nucleic Acid Protein Analyzer. The OD260/OD280 ratio was higher than 1.8, demonstrating that the extracted RNA is relatively pure and free of protein contaminants. Total RNA integrity was then detected: 1 μL of RNA sample was loaded onto 1% agarose gels, electrophoresis was run at 80 V for 20 min, and rRNA bands of total RNA were observed after 5, 18, and 28 s using a gel-imaging system (Tanon 1220, Shanghai Tianneng Technology Co., Ltd.). The completion of total RNA extraction can be verified when all three bands are present. Total RNA was reverse transcribed into cDNA using a reverse transcription kit (Promega). Based on the NCBI database sequence, primers in the experiment were synthesized by Shanghai Biotechnology Company. The primer sequences for the NMDAR NR1 subunit are as follows: CCTCAAGTCCCACGAGAATG (forward primer) and TCAAAAGTAAGGGTCGCAGG (reverse primer). For the 18s rRNA, the primer sequences are: CCTGGATACCGCAGCTAGGA (forward primer) and GCGGCGCAATACGAATGCCCC (reverse primer). Real-time PCR was conducted utilizing the ABI PRISM ^®^ 7500 Sequence Detection System (Applied Biosystems). The reaction conditions were pre-denaturation at 95°C for 4 min, 1 cycle; denaturation at 95°C for 15 s, annealing at 60°C for 32s, extending for 1 min at 70°C, 40 cycles, and extra-extension for 5 min at 72°C. At the end of the reaction, a melting curve was obtained for each sample and the relative expression level was calculated using the (2 ^–ΔΔ CT^) method.

### 2.4 Statistical analysis

Statistical analysis was performed using the software SPSS v27.0 (IBM Corp., Armonk, NY, USA). Continuous values are presented as means ± standard deviation if normally distributed; otherwise, they are presented as median (lower and upper quartiles) if non-normally distributed. Comparisons of variables between groups were performed using the t-test, and data that did not conform to the normal distribution were converted using the square root transformation to ensure the normality of distribution. Pearson’s or Spearman’s test was used for correlation analysis. We conducted receiver operating characteristic (ROC) analysis to evaluate the accuracy of Glu concentrations and the mRNA expression levels of the NMDAR NR1 subunit in distinguishing individuals with ID from HCs. The area under the ROC curve (area under the curve, AUC), sensitivity, and specificity were calculated to assess the estimation validity. The optimal value cutoff level was determined as the level that exhibited the highest sensitivity among the maximal values on the Youden index, calculated as (sensitivity + specificity) –1.

## 3 Results

### 3.1 Demographic and clinical features

We recruited 34 ID participants, 4 of whom were excluded from the analysis (2 were diagnosed with obstructive sleep apnea-hypopnea syndrome and 2 with periodic limb movement disorder). Finally, this study included 30 participants (mean age = 39.13 ± 11.97 years; 13 males) in the ID group and 30 participants (mean age = 34.67 ± 13.68 years; 13 males) in the HC group. Statistical analysis showed no significant differences in age (*P* = 0.182), gender (*P* = 0.759), marital status (*P* = 0.592), education (*P* = 0.101) and family history (*P* = 0.161) between the two groups. Among ID patients, the average age at first-onset was 35.10 ± 9.69 years, 21 patients were in the first episode, the time of current course was 12.50 (4.00–25.25) months, and the total duration of illness was 25.00 (12.75–79.50) months. Furthermore, there were significant differences between the two groups in the total scores of HAMA, HAMD-17, and PSQI and its subscale (sleep quality, sleep latency, sleep duration, habitual sleep efficiency, sleep disturbances, use of sleeping medication, daytime dysfunction) (**Table 1**).

**Table 1:** Demographic and clinical features of participants.

Characteristics	ID (N=30)	HC (N=30)	T or χ2	*P*
Number of subjects	30	30	–	–
Age(year)	39.13 ± 8.53	34.67 ± 9.05	1.346	0.182
Gender(male/female)	14/16	13/17	0.067	0.795
Married (yes/no)	20/10	18/12	0.287	0.592
Education (year)	14.03 ± 3.05	12.67 ± 3.29	1.669	0.101
Family history (yes/no)	4/26	1/29	1.964	0.161
First-episode (yes/no)	21/9	–	–	–
Age of first onset	35.10 ± 9.69	–	–	–
Time of current course (month)	12.50 (4.00–25.25)	–	–	–
Total duration of illness (month)	25.00 (12.75–79.50)	–	–	–
HAMA total scores	9.80 ± 2.93	4.40 ± 2.20	11.373	< 0.001
HAMD total scores	11.73 ± 3.14	6.07 ± 1.64	11.310	< 0.001
PSQI total scores	15.43 ± 2.40	3.67 ± 1.69	21.949	< 0.001
Sleep quality	2.70 ± 0.47	0.93 ± 0.58	12.960	< 0.001
Sleep latency	2.77 ± 0.43	0.40 ± 0.23	19.692	< 0.001
Sleep duration	1.00 ± 0.74	2.47 ± 0.82	7.264	< 0.001
Habitual sleep efficiency	2.60 ± 0.81	0.77 ± 0.50	10.491	< 0.001
Sleep disturbances	1.40 ± 0.68	0.03 ± 0.08	10.710	< 0.001
Use of sleeping medication	0.93 ± 0.143	0	4.474	< 0.001
Daytime dysfunction	0.53 ± 0.63	2.57 ± 0.58	12.035	< 0.001

Subjective sleep quality, sleep latency, sleep duration, habitual sleep efficiency, sleep disturbances, use of sleeping medication, and daytime dysfunction are components of the PSQI.

ID, insomnia disorder; HC, healthy control; PSQI, Pittsburgh Sleep Quality Index; HAMD-17, Hamilton Depression Scale-17; HAMA, Hamilton Anxiety Scale.

### 3.2 Comparison between ID and HC groups in Glu, Gln, GAD concentrations and mRNA expression levels of NMDAR NR1 subunit

**Figure 1:**
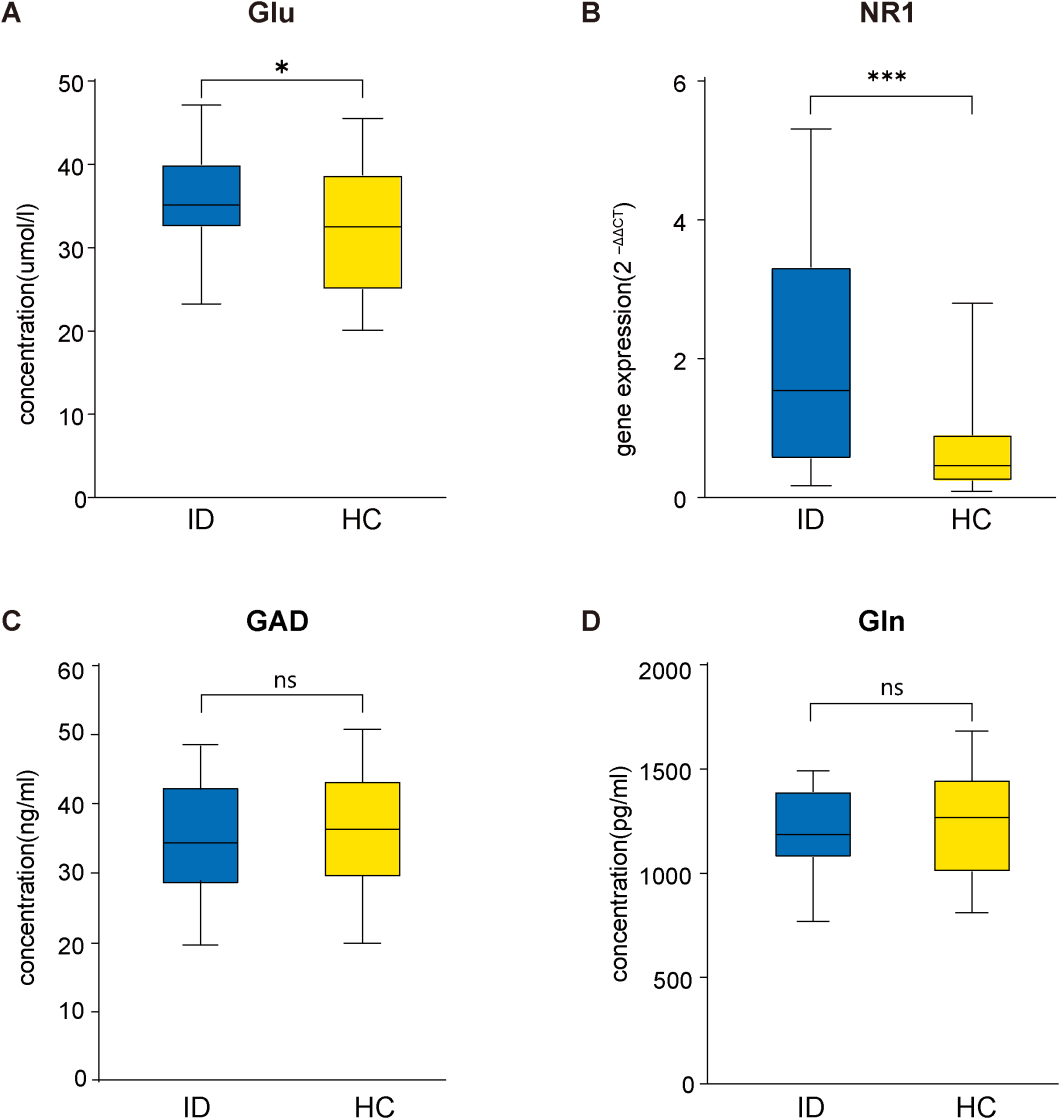
Comparison between ID and HC groups in Glu concentrations **(A)**, the mRNA expression levels of NMDAR NR1 subunit **(B)**, GAD concentrations **(C)** and Gln concentrations **(D)**. Glu, glutamate; NMDAR, Nmethyl-D-aspartate receptor; GAD, glutamic acid decarboxylase; Gln, glutamine; ID, insomnia disorder; HC, healthy control.

Compared with the HC group, the serum Glu concentrations (t = 2.089, *P* = 0.042) and the mRNA expression levels of the NMDAR NR1 subunit in peripheral blood (t = 4.245, *P* < 0.001) were significantly higher in the ID group (**Figure 1**). However, there was no significant difference in Gln (t = -0.632, *P* = 0.530) or GAD (t = -0.830, *P* = 0.410) between the ID group and HC group. In addition, Gln/Glu was significantly decreased (t = -2.318, *P* = 0.024) in the ID group compared with the HC group (**Supplementary Figure 1**).

### 3.3 ROC analysis for differentiation between the ID and HC groups

**Figure 2:**
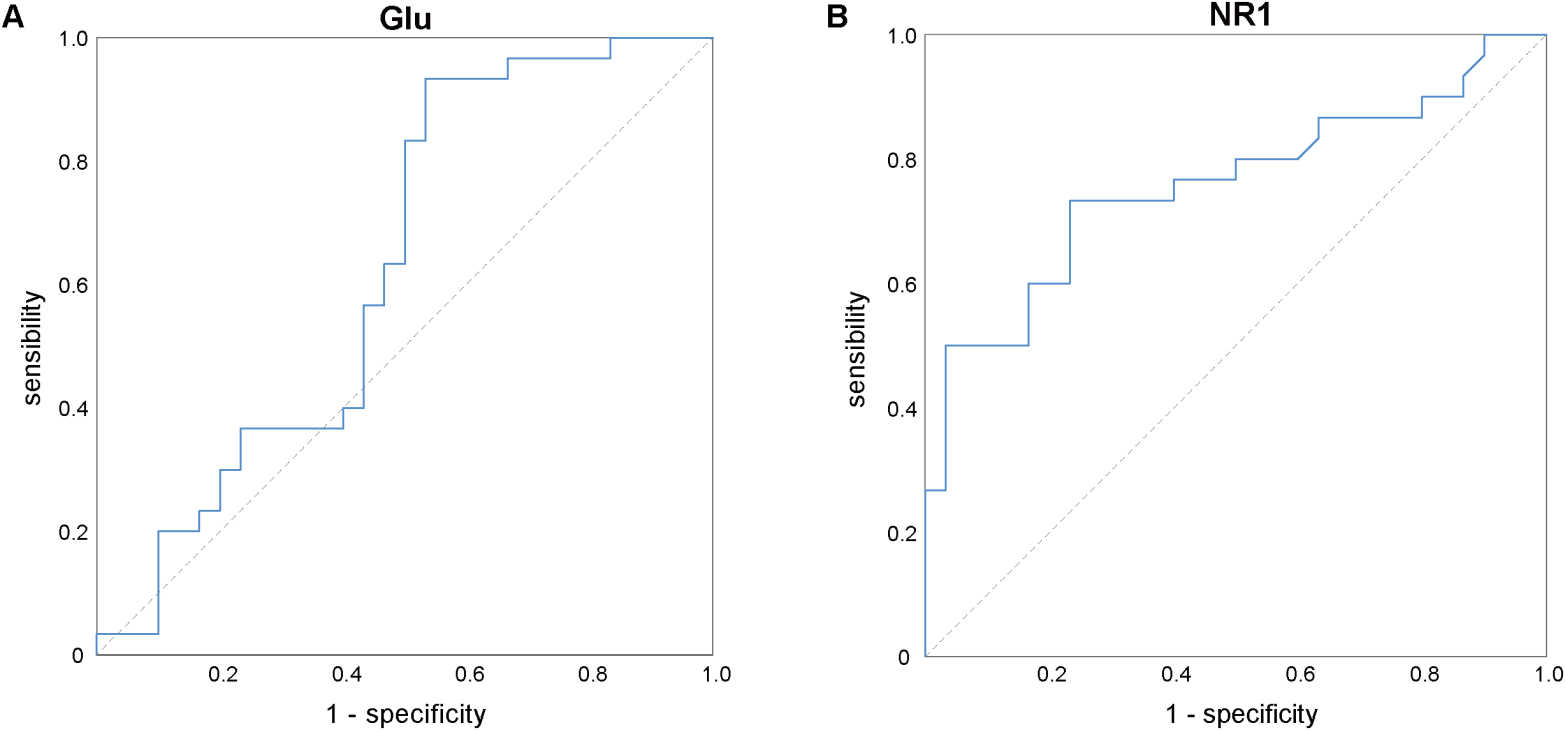
ROC analysis of Glu **(A)** and the mRNA expression levels of NMDAR NR1 subunit **(B)** for differentiation between the insomnia disorder and healthy control groups. Glu, glutamate; NMDAR, N-methyl-D-aspartate receptor.

The ROC curve (**Figure 2**) analysis indicated that the mRNA expression levels of the NMDAR NR1 subunit exhibited a moderate degree of validity in differentiating between ID patients and HCs (AUC = 0.758, *P* = 0.001, 95% confidence interval = 0.633–0.883). The optimal cutoff point for the mRNA expression levels of the NMDAR NR1 subunit was 0.86 (sensitivity = 73.3%, specificity = 76.7%). However, the Glu concentrations could not distinguish ID patients from HCs (AUC = 0.629, *P*= 0.086, 95% confidence interval = 0.483–0.775).

### 3.4 Association of Glu, Gln, GAD concentrations and mRNA expression levels of NMDAR NR1 subunit with clinical features in ID patients

No significant and qualitatively relevant correlations were found between the serum Glu, GAD concentrations and clinical features in the ID group. The mRNA expression levels of the NMDAR NR1 subunit in ID patients were found to be negatively correlated with age (r = −0.58, *P* < 0.001), total duration of illness (r = −0.42, *P* = 0.021), age of first onset (r = −0.57, *P* < 0.001) but positively correlated with daytime dysfunction (r = 0.45, *P* = 0.014). No statistically significant difference was found between the mRNA expression levels of the NMDAR NR1 subunit and gender, total disease duration, and PSQI total scores. And Gln was negatively correlated with total duration of illness (r=-0.435, *P*=0.016) (**Table 2**).

**Table 2:** Association of Glu concentrations and the mRNA expression levels of NMDAR NR1 subunit with clinical features in Insomnia disorder patients.

Characteristics	Glu		Gln		GAD		NR1 mRNA	
	r	*P*	r	*P*	r	*P*	r	*P*
Age	-0.125	0.509	-0.296	0.112	0.293	0.117	-0.58^***^	0.001
Total duration of illness	0.25	0.183	-0.435*	0.016	0.056	0.767	-0.42^*^	0.021
Age of first onset	0.01	0.097	-0.159	0.402	0.323	0.081	-0.57^**^	0.001
HAMA	0.28	0.141	-0.308	0.098	0.212	0.261	0.01	0.962
HAMD	-0.22	0.251	-0.226	0.230	0.196	0.298	0.09	0.651
PSQI	0.09	0.652	-0.077	0.686	0.209	0.267	-0.14	0.472
Subjective sleep quality	0.05	0.791	0.080	0.673	0.256	0.172	0.03	0.877
Sleep latency	-0.21	0.266	-0.026	0.891	0.224	0.234	-0.42^*^	0.021
Sleep duration	-0.31	0.479	0.018	0.924	0.029	0.881	0.21	0.263
Habitual sleep efficiency	-0.22	0.240	0.064	0.736	0.026	0.892	-0.18	0.346
Sleep disturbances	-0.25	0.177	0.244	0.194	0.151	0.425	-0.13	0.505
Use of sleeping medication	-0.21	0.270	-0.395	0.091	0.155	0.413	-0.31	0.100
Daytime dysfunction	0.11	0.573	0.040	0.833	0.022	0.909	0.45^*^	0.014

*P < 0.05; ***P < 0.001. Subjective sleep quality, sleep latency, sleep duration, habitual sleep efficiency, sleep disturbances, use of sleeping medication, and daytime dysfunction are components of the PSQI.

Glu, glutamate; Gln, glutamine; GAD, glutamic acid decarboxylase; NR1, the mRNA expression levels of NMDAR NR1 subunit; NMDAR, N-methyl-D-aspartate receptor; PSQI, Pittsburgh Sleep Quality Index; HAMD-17, Hamilton Depression Scale-17; HAMA, Hamilton Anxiety Scale.

### 3.5 The correlation analysis of serum Glu, Gln, and GAD

**Figure 3:**
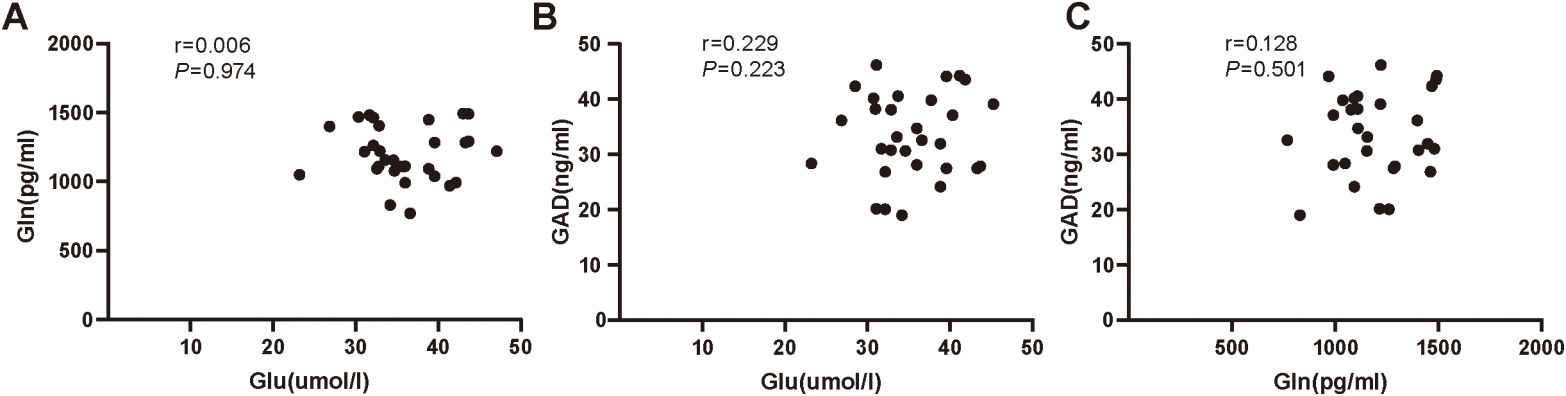
**(A)** The correlation analysis of serum Glu and Gln in insomnia disorder group. **(B)** The correlation analysis of serum Glu and GAD in insomnia disorder group. **(C)** The correlation analysis of serum Gln and GAD in insomnia disorder group. Glu, glutamate; Gln, glutamine; GAD, glutamic acid decarboxylase.

**Figure 4:**
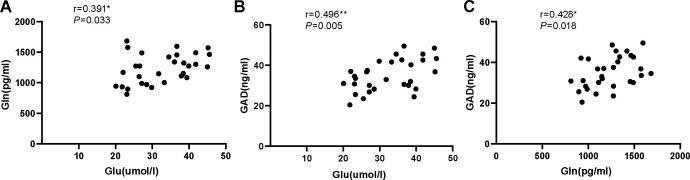
**(A)** The correlation analysis of serum Glu and Gln in healthy control group. **(B)** The correlation analysis of serum Glu and GAD in healthy control group. **(C)** The correlation analysis of serum Gln and GAD in healthy control group. Glu, glutamate; Gln, glutamine; GAD, glutamic acid decarboxylase.

Our results found no significant correlation between serum levels of Glu and Gln (r = 0.006, *P*=0.974), serum levels of GAD and Glu (r=0.229, *P*=0.223), or serum levels of GAD and Gln (r=0.128, *P*=0.501) in the ID group (**Figure 3**). However, significant correlations were observed between serum levels of Glu and Gln (r=0.391, *P*=0.033), serum levels of GAD and Glu (r=0.496, *P*=0.005), and serum levels of GAD and Gln (r=0.428, *P*=0.018) in the HC group (**Figure 4**).

## 4 Discussion

We assessed the serum Glu concentrations and mRNA expression levels of the NMDAR NR1 subunit in ID patients to evaluate the potential of these peripheral measures as biomarkers for ID. We found that the serum Glu concentrations and mRNA expression levels of NMDAR NR1 subunit in ID patients were higher than those in the control group, suggesting the dysregulation of glutamatergic pathways in ID. To our knowledge, this is the first study revealing upregulated expression of NMDAR NR1 subunit in patients with ID.

Glu is the most abundant amino acid neurotransmitter in the brain, which can partially enter the peripheral blood circulation and exchange information bi-directionally (32). Researchers have investigated the Glu levels of the CNS in ID patients. MRS-based studies indicated that Glu levels in basal ganglia were increased after sleep loss (36, 37). However, several previous studies using MRS have shown no difference in Glu levels in the left occipital cortex, left prefrontal cortex, anterior cingulate cortex, and thalamus regions of the brain between ID patients and controls (17–19). Glu exhibits varying associations with sleep/wake stages across diverse brain regions (38). The reason for the distinction can be associated with the specificity of brain regions and the heterogeneity of samples of ID patients. ID patients showed no differences in brain Gln and Glx levels compared to the control group (18). Because Glx comprises both Glu and Gln signals, studies indicating increased Glx could indicate elevated levels of Glu, Gln, or both metabolites concurrently (39). When analyzing peripheral Glu, a study found that ID patients had increased serum Glu levels compared with healthy people, which is consistent with our study (40). However, there have been no studies to measure Gln levels in peripheral blood.

The NMDAR exists in CNS and peripheral organs and exhibits unique properties that play an indispensable role in sleep, emotion, learning, and memory, but overstimulation of NMDAR induces several signal cascades leading to cell apoptosis (41, 42). NMDAR comprises NR1, NR2, and NR3 subunits; among these subunits, NR1 is the fundamental component (43). NMDAR activation regulates the rhythms of sleep and is required for various sleep properties (29, 44). Changes in the expression of the NMDAR NR1 subunit in ID patients have not been reported. A molecular imaging study demonstrated that the availability of cerebral functional metabotropic Glu receptors of subtype 5 (mGluR5) was increased after sleep deprivation (45). In an animal experiment, the surface expression level of NMDAR NR1 within the hippocampus was upregulated in mice with longer deprivation (SD) compared with mice with shorter SD (46). Another study with rats observed increased protein levels of the NMDAR NR1 subunit following treatment with sedative-hypnotic (47). Our study found that NMDAR NR1 subunit was upregulated in ID patients, which was similar to these findings, suggesting that ID was linked to the change in expression of Glu receptor.

Moreover, ROC analysis suggested that the mRNA expression levels of the NMDAR NR1 subunit in peripheral blood may be a potential biomarker to help diagnose ID. To our knowledge, the present study is the first to demonstrate an increase in the mRNA expression levels of NMDAR NR1 subunit in ID people. Blood-based biomarkers have proven beneficial in clinical practice in other medical specialties because of the ease of access and low invasiveness of the bio-sampling method (48). While measuring neurotransmitter concentrations or their receptor expression levels in cerebrospinal fluid is ideal, obtaining individual samples may present challenges (49). The availability of biomarker tests would allow for earlier diagnosis, improved diagnostic accuracy, and permit the stratification of patients for more effective treatment plans.

It is not entirely clear how the peripheral Glu, Gln or the mRNA expression levels of NMDAR NR1 subunit reflect CNS levels. Some studies indicate a correlation of glutamatergic neurotransmission between the CNS and the periphery. For instance, Rollins et al. demonstrated that mRNA transcripts in blood reflect approximately 20% of the transcripts expressed in brain tissues (50). And a positive correlation was found between serum and cerebrospinal fluid concentrations of Glu in 10 healthy people (51). Interactions between the brain and periphery occur in diverse ways, including neurotransmitters and soluble receptors (52, 53). Additionally, elevated extracellular glutamate is considered to trigger the opening of BBB, and NMDAR activation damages mucosal barrier integrity, thereby increasing BBB permeability (54, 55). Because of the communication between CNS and non-neuronal cells, Glu concentrations and receptor expression in peripheral blood may indicate pathophysiological changes in the CNS. However, more research is needed to comprehend the correlation of glutamatergic neurotransmission between the CNS and the periphery.

The present study found a negative correlation between the mRNA expression levels of the NMDAR NR1 subunit and age, total duration of illness, and age of first onset in ID patients, which may indicate that the Glu activation response in ID patients diminishes with increasing age and disease duration. Previous studies have reported that the expression of NMDAR declines with age (56–59). For example, an animal model revealed that the density of NR2b immunoreactive cells within the anteroventral periventricular decreased as a function of age (59). Since insomnia symptoms are considered to be an age-related process (60), the above finding implies that age may influence the occurrence of ID by affecting NMDAR expression; however, further research is needed to elucidate this relationship. In adult rats, NMDAR expression in the hippocampus was upregulated in the early phase and downregulated in the late phase following NMDAR antagonist administration (61). Additionally, patients with anti-NMDAR encephalitis showed a correlation between hippocampal volumetry and disease duration (62). Based on these studies and our results, we speculate that illness duration influences NMDAR expression in a certain way. The NR1 subunit is a critical component of the NMDA receptor, and thus NR1 subunit mRNA expression reflects NMDA receptor expression levels. We also found a positive correlation between the mRNA expression levels of the NMDAR NR1 subunit and daytime dysfunction. Although there are no existing reports on this correlation, it is hypothesized that the observed positive correlation might be due to compensatory mechanisms in ID, given that NMDAR activation plays a role in promoting wakefulness (28).

In addition, our results found no significant correlation between serum levels of Glu and Gln, serum levels of GAD and Glu, or serum levels of GAD and Gln in the ID group, but significant correlations were observed between serum levels of Glu and Gln, serum levels of GAD and Glu, and serum levels of GAD and Gln in the HC group. This may indicate a dysregulation in the metabolism of glutamatergic neurotransmission in ID, providing a novel perspective on the pathophysiology of ID.

This study has some limitations. First, we did not simultaneously investigate changes of glutamatergic neurotransmission in the CNS and peripheral blood. Further experimentation should be required to determine if co-regulation of altered blood and brain expression is found in ID. Second, this study examined only the mRNA expression levels of NMDAR NR1 subunit in ion channel Glu receptors and failed to detect the expression of metabolic Glu receptors simultaneously. Thirdly, the sample size is relatively small; thus, future studies should aim to expand the sample size to enhance the persuasiveness of the findings.

## 5 Conclusion

The present study suggested that glutamatergic neurotransmission was abnormal in ID patients, which may play a critical role in the pathogenesis of ID. Additionally, the mRNA expression levels of the NMDAR NR1 subunit appeared to have potential as a clinical biomarker for ID. However, the sample size of our study was limited, which may affect the generalizability of the results. Therefore, future studies with larger sample sizes are needed to further validate and explore the mechanisms involved and to assess the reliability of the biomarker.

## Data Availability

The original contributions presented in the study are included in the article/supplementary material. Further inquiries can be directed to the corresponding authors.
