# Supplementary material for: Elevated peripheral glutamate and upregulated expression of NMDA receptor NR1 subunit in insomnia disorder
Source: Front Psychiatry. 2024 Oct 7;15:1436024. doi: 10.3389/fpsyt.2024.1436024 (PMC11491378; doi:10.3389/fpsyt.2024.1436024)
Supplement: Supplementary file 1 [file DataSheet1.docx]

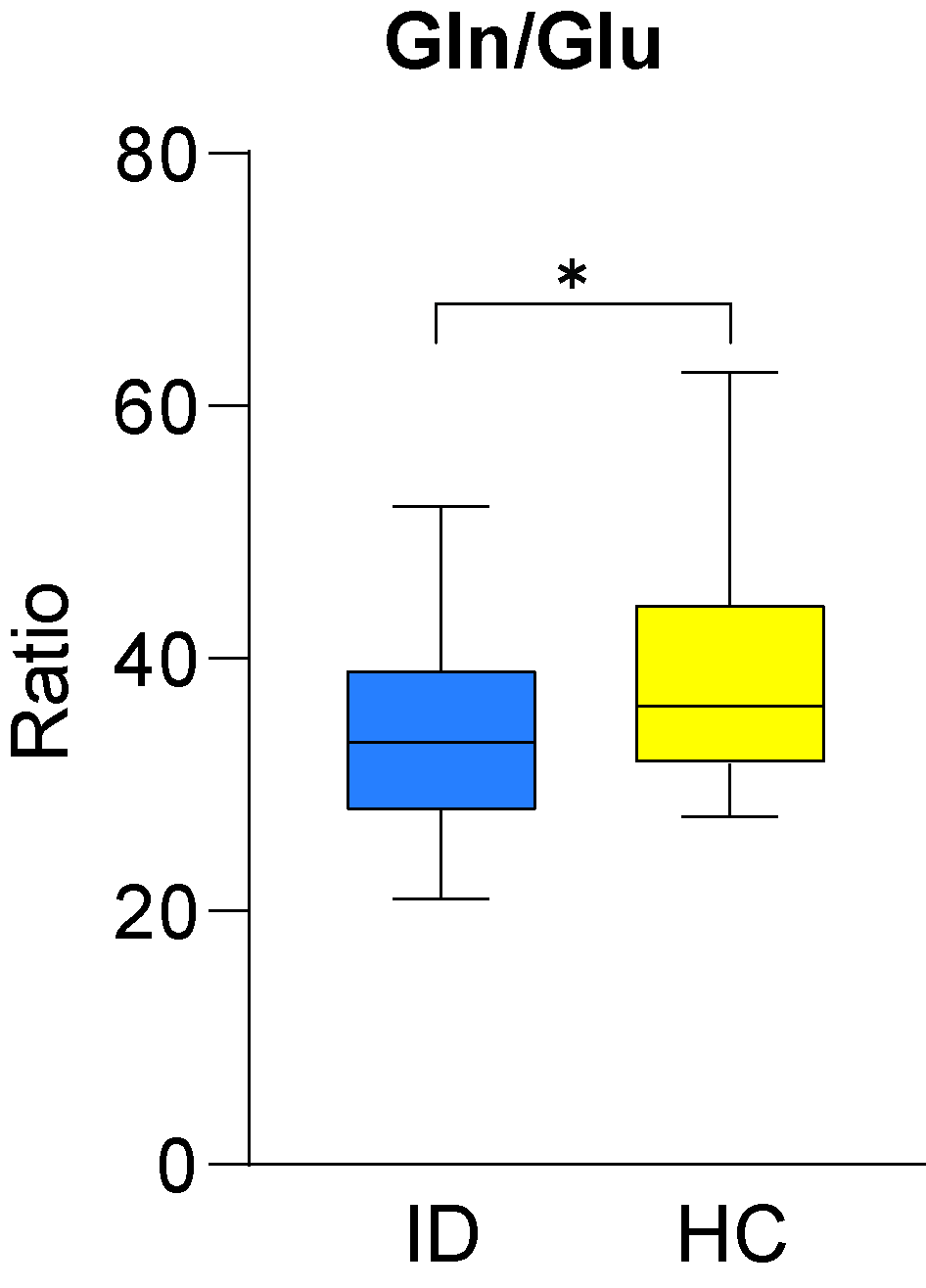


Supplementary Figure 1. Gln/Glu was significantly decreased (t = -2.318, P = 0.024) in the insomnia disorder group. Glu, glutamate; Gln, glutamine.
